# Complete Genome Sequence of Biocontrol Strain *Pseudomonas fluorescens* LBUM223

**DOI:** 10.1128/genomeA.00443-15

**Published:** 2015-05-07

**Authors:** Roxane Roquigny, Tanya Arseneault, Vijay J. Gadkar, Amy Novinscak, David L. Joly, Martin Filion

**Affiliations:** Department of Biology, Université de Moncton, Moncton, New Brunswick, Canada

## Abstract

*Pseudomonas fluorescens* LBUM223 is a plant growth-promoting rhizobacterium (PGPR) with biocontrol activity against various plant pathogens. It produces the antimicrobial metabolite phenazine-1-carboxylic acid, which is involved in the biocontrol of *Streptomyces scabies*, the causal agent of common scab of potato. Here, we report the complete genome sequence of *P. fluorescens* LBUM223.

## GENOME ANNOUNCEMENT

*Pseudomonas fluorescens* LBUM223 is a Gram-negative, rod-shaped bacterium with beneficial plant growth-promoting and biocontrol activities which was isolated from the rhizosphere of a strawberry plant in eastern Canada (1–4). It is capable of promoting the growth of potatoes (1) and inhibiting the growth of several plant pathogens (4). Its ability to produce phenazine-1-carboxylic acid (PCA) has been shown to be involved in the biocontrol of common scab of potato, a disease caused by the bacterial plant pathogen *Streptomyces scabies* (1, 2). Several studies have also demonstrated that phenazines are secondary metabolites that play a major role in the biological control of various plant pathogens (5, 6).

Genomic DNA was extracted using the UltraClean microbial DNA isolation kit (Mo Bio, Carlsbad, CA, USA) and purified using Agencourt AMPure XP beads (Beckman Coulter, Mississauga, Canada), according to the manufacturer’s instructions. The genome of LBUM223 was sequenced using PacBio single-molecule real-time (SMRT) sequencing technology (McGill University and Génome Québec Innovation Centre, Montreal, Canada), generating a total of 176,424 raw subreads, with an average length of 5,556 bp. Three single-molecule real-time (SMRT) DNA sequencing cells were used in a PacBio RSII sequencer. Genome assembly was performed using the Hierarchical Genome Assembly Process (HGAP), generating a circular 6,694,199-bp chromosome with 59.4% G+C content. No functional plasmid was detected. The RAST server (7) was used to predict and annotate 5,966 protein-coding genes, 68 tRNA genes, and 6 rRNA operons.

Ten core housekeeping genes (*acsA*, *aroE*, *dnaE*, *guaA*, *gyrB*, *mutL*, *ppsA*, *pyrC*, *recA*, and *rpoB* [8]) from 48 closely related *Pseudomonas* spp. were retrieved from GenBank and compared based on concatenated alignments using the CLC Genomics Workbench software version 8.0 (CLC bio, Boston, MA). The resulting maximum-likelihood phylogenetic tree indicated that *P. fluorescens* LBUM223 belongs to *P. fluorescens* subclade 3, according to Loper et al. (8). Within this subclade, *P. fluorescens* A506 (complete genome sequence accession no. CP003041) was identified as the closest relative of LBUM223. Genome analysis of *P. fluorescens* LBUM223 showed that this strain possesses one copy of the seven-gene *phzABCDEFG* operon responsible for the biosynthesis of PCA. Furthermore, genes involved in 1-aminocyclopropane-1-carboxylate deaminase (*acdS*) and pyrroloquinoline-quinone (*pqqABCDEF*) production, potentially associated with plant growth-promoting activity (8, 9), were detected in the genome of LBUM223. It also possesses several genes associated with type I (*lap* cluster), type II (*tad* cluster), and type VI (*imp* cluster) secretion systems, as well as biosynthetic and membrane receptor genes associated with pyoverdine production and uptake.

### Nucleotide sequence accession numbers. 

This complete genome project has been deposited in DDBJ/ENA/GenBank under the accession no. CP011117. The version described in this paper is the first version, CP011117.1.
